# Efficacy of Ziprasidone in Controlling Agitation during Post-Traumatic Amnesia

**DOI:** 10.1155/2007/529076

**Published:** 2007-02-12

**Authors:** Enrique Noé, Joan Ferri, Carlos Trénor, Javier Chirivella

**Affiliations:** Servicio de Daño Cerebral de Hospitales NISAInstituto Valenciano de Neurorrehabilitación (IVAN)ValenciaSpain

**Keywords:** Posttraumatic amnesia, traumatic brain injury, ziprasidone, neuroleptics, agitation

## Abstract

*Objectives:* to provide our initial experience with ziprasidone in the management of behaviour problems of patients with traumatic brain injury (TBI) during the period of post-traumatic amnesia (PTA). *Patients:* Five patients with a mean age of 26.8 ± 9.8 years who had suffered a severe TBI (Glasgow Coma Scale score < 8; length of coma: 16 ± 7.1 days; and length of PTA: 62.4 ± 14.8 days) were included in the study. Agitation was assessed by the Agitated Behaviour Scale (ABS) prior to the administration of ziprasidone, after two weeks of initiating treatment and at the moment of discontinuing ziprasidone. *Results:* ABS Total score decreased from 27.2 ± 3 to 18 ± 1.2 after two weeks of treatment. The same decrease was also noticed in each one of the subscales (dishinhibition, aggressiveness and lability). Mean dose of the drug was 52.8 ± 27.11 mg/day (range: 20–80 mg), with the highest dose ranging between 40 and 80 mg (64 ± 21.9 mg). Maximum period of administration of ziprasidone was 48.2 ± 14.8 days (range: 35–68 days). No clinical or electrocardiographic side effects were reported. *Conclusion: *This study shows the efficacy of ziprasidone in controlling agitation during the PTA period. Despite the small size of our sample, ziprasidone reduced symptoms of agitation quickly and with good tolerability, safety and no side effects.

## 1. Introduction

The period of post-traumatic amnesia (PTA) is one of the clinical variables with more prognostic value to be considered after traumatic brain injury (TBI). From a neuropsychological point of view, this period of time is characterized by time-space disorientation and inability to learn material from daily living. Also, usually during this phase, behaviour alterations are frequent. Recently, these alterations have been estimated to happen between 11 and 50% of the TBI, depending on their severity, being akathisia, anger, disinhibition and emotional labilitiy the predominant symptoms [1, 2,8,14]. Although the cause of these alterations is not completely clarified it seems that they are related to the intense lack of self-awareness associated with memory alterations so characteristic of the initial stages after a TBI. This cognitive alteration would prevent the patient from accurately perceiving their situation and environment, leading to inappropriate responses to stimuli.

Traditionally, "classic or typical" neuroleptics have been one of the most common kinds of drugs employed for the treatment of behaviour problems occurring after a TBI, along with benzodiazepines, antiepileptic, beta-blockers and antidepressants drugs, among others [1, 13,15]. During the last few years, with the appearance of the "atypical" neuroleptics, we count on a new therapeutic tool with a very specific profile to treat this kind of pathology. These new drugs, unlike the typical ones, don't have the negative effects on cognitive and motor functions and show also an indirect antidepressant effect. All these qualities place, at least from a theoretical point of view, these new neuroleptics as the first therapeutic option for the treatment of agitation associated to severe TBI.

Ziprasidone is one of the most recently commercialized "atypical" neuroleptic. This drug binds to

ISSN 0953-4180/07/$17.00 © 2007 – IOS Press and the authors. All rights reserved dopaminergic and serotoninergic receptors and inhibits serotonine/norepinephrine recaptation, which may explain its antidepressant effect [19]. Furthermore, it has an alfa-1 effect, making necessary be aware of the possibility of tachycardia and hypotension. This effect added to the fact that it may increase the QT interval, have raised concerns about its safety in patients with arrhythmias, previous prolonged QT, recent myocardial infarction, cardiac insufficiency or hydroelectrolitic alterations. Ziprasidone may be administered orally or by intramuscular injection, which facilitates a relatively fast onset of action, being of special usefulness in emergency situations.

Treatment with ziprasidone has proven to be efficacious in the management of acute exacerbations of schizophrenic patients and schizoaffective disorders, as in other neurological disorders such as Tourette Syndrome [6,10,16,19]. The aim of our study is to provide our initial experience with this drug in the management of behaviour problems of patients with TBI during the period of PTA. To date; there are no previous publications, that we are aware of, demonstrating the efficacy of ziprasidone in this kind of pathology during this particular period of time.

## 2. Method

### *2.1. Patients*

Patients were selected using a pre-existing database including all patients attending our facility during 2003. The initial population comprised the database of 37 patients who had sustained a severe head injury. From the patient database, subjects were selected according to the following criteria. Patients were included in the study if they were at the stage of post-traumatic amnesia at admission, received treatment with ziprasidone and were out of post-traumatic amnesia at the time of finishing the study (December 2003). A total of seven patients who fulfilled the inclusion criteria were identified by a retrospective analysis performed through our database. Three additional patients treated with ziprasidone were excluded since they presented severe cognitive impairment and they were still in PTA by the date of submitting this paper. These three patients are receiving ziprasidone with good results and tolerance but the attempts to withdrawal have been associated with an increase in agitation not acceptable to their families. From the seven patients initially detected, one of them could not be accurately followed up since he moved to another place and voluntarily discharged himself from the hospital. A second patient was excluded since he presented premorbid schizophrenia, which had been neither diagnosed nor treated (TBI was the result of a suicide attempt during a psychotic episode).

### *2.2. Assessment protocol*

All patients were evaluated by a wide assessment protocol that included clinical, demographic and TBI related variables. Evaluation of the PTA was performed prospectively by daily application of the Galveston Test of Orientation and Amnesia to each patient since the moment of admission to our Service. According to the normative scale of this test, it was considered that a patient was out of the PTA if the score obtained on three consecutive days was equal or superior to 75. Length of the PTA was calculated by subtracting the period in coma estimated for each subject (Length of PTA = date of end PTA − date of end of coma). Since severe cognitive impairment characterizes PTA period, neuropsychological evaluation was assessed with the Spanish version of the Severe Impairment Battery (SIB) at the beginning of treatment and once PTA was resolved [9]. This scale was designed to evaluate different cognitive abilities (social interaction, orientation, attention, memory, praxis, visuospatial and constructional abilities, and language) of severely impaired patients. The SIB is composed of 40 simple, one-step command items that are quick to score on a three-point scale. Overall scores can range from 0–100 allowing for categorization of degree of impairment.

Agitation was assessed by the Agitated Behaviour Scale prior to the administration of ziprasidone, after two weeks of treatment and at the moment of discontinuing ziprasidone. Fourteen items assessing different noticeable aspects of the agitation episodes compose this scale. This items range from cognitive symptoms (distractibility, inability to concentrate, impulsiveness) to behavioural (verbal or physical aggressiveness, repetitive motor acts, etc.) and emotional aspects (inappropriate laughter or crying, quick mood changes, etc.). Each item is scored with a Likert scale that ranges between 1 (absence of behaviour) and 4 (presence of extreme intensity behaviour), the lowest score obtained being 14 and the highest 56. The scoring on the scale was performed globally and divided into three different subscales (disinhibition, agressiveness and lability) according to the norms of correction and scoring of the scale.

Table 1
Demographical data. Absolute values and mean ± standard deviation

| | P1 | P2 | P3 | P4 | P5 | Total |
|---|---|---|---|---|---|---|
| Age (years) | 17 | 41 | 18 | 30 | 28 | 26.8 ± 9.8 |
| Gender (Male/Female) | Male | Male | Male | Female | Female | – |
| Premorbid IQ | 115 | 111 | 112 | 121 | 134 | 118.6 ± 9.4 |
| Schooling (years) | 12 | 10 | 13 | 12 | 16 | 12.6 ± 2.1 |
| Glasgow (3–15) | 8 | 4 | 8 | 6 | 4 | – |
| DRS (at admission) | 9 | 9 | 15 | 5 | 15 | 10.6 ± 4.3 |
| SIB (at admission) | 92 | 90 | 96 | 98 | 92 | 93.6 ± 3.2 |
| SIB (after PTA resolution) | 100 | 100 | 100 | 100 | 100 | 100 |
| Length of Coma (days) | 15 | 4 | 20 | 21 | 20 | 16 ± 7.1 |
| Length of PTA (days) | 75 | 80 | 60 | 52 | 45 | 62.4 ± 14.8 |

IQ: Intelligence Quotient. DRS: Disability Rating Scale. PTA: Post-traumatic Amnesia. SIB: Severe Impairment Battery.

Assessment of side effects was performed weekly in a semi-structured way. Specifically, an electrocardiogram (EKG) was performed at the beginning and after two weeks of therapy with ziprasidone to detect possible electrocardiographic alterations.

## 3. Results

### 3.1. Demographic and TBI related variables

A total of 5 patients participated in this study (three males and two females), mean age 26.8 ± 9.8 years. Clinical and demographic characteristics of the patients are shown in Table 1. All patients had suffered a severe TBI according to their Glasgow Coma Scale scores (< 8 in every case), length of coma period (16 ± 7.1 days) and length of PTA period (62.4 ± 14.8 days). Mean years of formal education were high (12.6 ± 2.1 years) as was their estimated premorbid Intelligence Quotient (118.6 ± 9.4). Given that at the time of admission all patients were at the PTA period and that this phase is characterized by a profound cognitive impairment, the degree of dependence in our sample assessed by the Disability Rating Scale, was high in all cases (10.6 ± 4.3, range: 5–15). Cognitive impairment, assessed with the SIB showed scores close to maximum range in all patients (93.6 ± 3.2).

### 3.2. Clinical variables

For all patients, the onset of therapy with ziprasidone coincided with the moment of admission to our Service, with a mean of 54.6 ± 11.5 days after the TBI (range: 40–72 days). Mean dose of the drug (daily dose in mg/number of days of therapy) was 52.8 ± 27.11 mg/day (range: 20–80 mg), with the highest dose ranging between 40 and 80 mg (64 ± 21.9 mg). Maximum duration of administration of ziprasidone ranged between 35 and 68 days, mean 48.2 ± 14.8 days (Table 2).

Table 2 shows partial and total values (disinhibition, lability and aggressiveness) from the Agitation Scale at the beginning and two weeks after ziprasidone therapy. Total score decreased from 27.2 ± 3 to 18 ± 1.2 at the two-week interval. The same decrease was also noticed in each one of the subscales. In order of intensity, this decrease was higher in disinhibition (28.6 ± 2 initial versus 17.1 ± 1.4 final) followed by aggressiveness (24.5 ± 10.5 initial versus 16.8 ± 2.9 final), with the minor effect observed on the items directed to assess lability (26.1 ± 6.2 initial versus 20.4 ± 2.5 final). At the moment of ziprasidone withdrawal all patients scored 1 (absence of behaviour) in each item of that scale.

All patients tolerated ziprasidone from the beginning of the therapy with no clinical or electrocardiographic side effects.

## 4. Discussion

This study shows the efficacy of ziprasidone in controlling agitation during the PTA period. Despite the small size of our sample, ziprasidone reduced symptoms of agitation quickly and with good tolerability, safety and no side effects.

Classically the use of neuroleptics in patients suffering from TBI has not been recommended for the recovery of motor and cognitive functions due to their negative side effects. It has been accepted that the new atypical neuroleptics show a pharmacologic profile much more attractive than the classic ones. In spite of this generalized opinion on behalf of the atypical neuroleptics to control agitation occurring after a TBI,

Table 2
Clinical variables. Absolute values and mean ± standard deviation

| | P1 | P2 | P3 | P4 | P5 | Total |
|---|---|---|---|---|---|---|
| Maximum dose (mg/day) | 80 | 80 | 40 | 40 | 80 | 64 ± 21.9 |
| Days of treatment with ziprasidone | 35 | 41 | 68 | 37 | 60 | 48.2 ± 14.8 |
| Mean dose of ziprasidone (mg/day) | 20 | 80 | 30 | 57.2 | 77 | 52.8 ± 27.1 |
| Beginning ziprasidone (days after TBI) | 72 | 40 | 54 | 51 | 56 | 54.6 ± 11.5 |
| *Initial Agitation Scale* | | | | | | |
| Total | 31 | 29 | 27 | 23 | 26 | 27.2 ± 3 |
| Disinhibition | 28 | 29.7 | 28 | 26.2 | 31.5 | 28.6 ± 2 |
| Aggressiveness | 35 | 35 | 24.5 | 14 | 14 | 24.5 ± 10.5 |
| Lability | 37.7 | 23.3 | 23.3 | 23.3 | 23.3 | 26.1 ± 6.2 |
| *Agitation Scale* (*14 days of Treatment*) | | | | | | |
| Total | 19 | 18 | 19 | 16 | 18 | 18 ± 1.2 |
| Disinhibition | 17.5 | 15.7 | 17.5 | 15.7 | 19.2 | 17.1 ± 1.4 |
| Aggressiveness | 17.5 | 21 | 17.5 | 14 | 14 | 16.8 ± 2.9 |
| Lability | 23.3 | 18.6 | 23.3 | 18.6 | 18.6 | 20.4 ± 11.5 |

studies dealing with this matter are scarce. The oldest of this kind of drugs, clozapine, has proved to be so useful to in controlling verbal/physical aggressiveness and certain bizarre motor behaviours in post-traumatic patients [11]. The risk of agranulocytosis (1% of patients on clozapine), the possible negative effects on mnesic functions related to its anticholinergic effect and the appearance of new drugs with a much safer profile, have left clozapine as a second drug of choice as antipsychotic in this and other pathologies [4]. Similarly, several case-reports have been published, showing the benefit of risperidone to regulate sleep pattern and psychotic symptoms as well as to control the eating disorders and motor restlessness derived from acquired brain injury [12,17,18]. Olanzapine has been used in patients who suffered TBI, as antipsychotic and mood stabilizer, but similarly to the rest of antipsychotic drugs, there are no well designed randomized trials demonstrating its efficacy in such pathology and, as well as with the other antipsychotics, only case-reports or small sized samples studies have been published [3,5]. To date, there are no reports describing the benefit of quetiapine in subjects with TBI and just one report showed the usefulness of loxapine for the treatment of one patient with olanzapine-resistant post-traumatic delirium [7].

Regarding ziprasidone, to our knowledge there are no published reports, demonstrating its efficacy in patients with TBI, and specifically for the treatment of post-traumatic agitation. Patients with TBI may show, mainly during the stage of post-traumatic amnesia, a wide variety of psychopathological symptoms (mood disorders, delusions, hallucinations, etc.). Given that ziprasidone has proved its efficacy for the treatment of agitation and psychotic symptoms, it could be expected to be useful to control post-traumatic agitation [6].

Characteristically, ziprasidone improved cognitive elements (lack of attention, inability to concentrate, impulsivity) as well as behavioural (verbal or physical aggressiveness, repetitive motor acts, etc.) and emotional ones. All patients improved cognitively during the follow-up period, provided that they came out from the post-traumatic amnesia period and their SIB scores reached normal ranges. However, the absence of a control group did not allow us to evaluate to what degree the treatment with ziprasidone could have altered the spontaneous recovery that takes place during the first months after a TBI. Moreover, it is not clear if the beneficial cognitive effects observed after therapy with ziprasidone are directly related to the action of the drug on determined neuropsychological functions, given its neurochemical affinity to receptors from specific neurological pathways implicated in cognitive processes, or they are derived from the reduction of psychopathological symptoms that characterize the stage of PTA.

The majority of our patients required an initial dose of 80 mg, in agreement with the dosage recommendations for this drug, although their maintenance dose was slightly inferior to the dosage suggested to treat other disorders such as schizophrenia. Characteristically, the benefit of therapy with ziprasidone was achieved fast, as previously described for schizophrenia or bipolar disorder, being noticeable from the beginning of therapy, which led to the reduction of agitation to almost normal levels after two weeks.

In sum, while this study is clearly limited by the small number of subjects and other limitations derived from case-report studies (fail to control for the effects of spontaneous recovery, placebo effects, etc.), the finding of improvement on a measure of agitation after ziprasidone therapy warrants further investigation based on large randomized clinical trials.

## References

[1] M.M. Brooke, D.R. Patterson, K.A. Questad, D. Cardenas and L. Farrel-Roberts, The treatment of agitation during initial hospitalization after traumatic brain injury, *Arch Phys Med Rehabil* **73** (1992), 917–921.

[2] M.M. Brooke, K.A. Questad, D.R. Patterson and K.J. Bashak, Agitation and restlessness after closed head injury: a prospective study of 100 consecutive admissions, *Arch Phys Med Rehabil* **73** (1992), 320–323.

[3] P.V. Butler, Diurnal variation in Cotard's syndrome (copresent with Capgras delusion) following traumatic brain injury, *Aust N Z J Psychiatry* **34** (2000), 684–687.

[4] T.E. Goldberg and D.R. Weinberger, The effects of clozapine on neurocognition: an overview, *J Clin Psychiatry* **55**(Suppl B) (1994), 88–90.

[5] T.W. Heinrich and J.T. Junig, Recurrent mania associated with repeated brain injury, *Gen Hosp Psychiatry* **26** (2004), 490–492.

[6] P.E. Keck, Jr., S.L. McElroy and L.M. Arnold, Ziprasidone: a new atypical antipsychotic, *Expert Opin Pharmacother* **2** (2001), 1033–1042.

[7] D. Krieger, K. Hansen, C. McDermott, R. Matthews, R. Mitchell, N. Bollegala et al., Loxapine versus olanzapine in the treatment of delirium following traumatic brain injury, *NeuroRehabilitation* **18** (2003), 205–208.

[8] H.S. Levin and R.G. Grossman, Behavioral sequelae of closed head injury. A quantitative study, *Arch Neurol* **35** (1978), 720–727.

[9] J. Llinas Regla, M. Lozano Gallego, O.L. Lopez, M. Gudayol Portabella, S. Lopez-Pousa, J. Vilalta Franch et al., Validation of the Spanish version of the Severe Impairment Battery, *Neurologia* **10** (1995), 14–18.

[10] L.S. Matza, T.M. Baker and D.A. Revicki, Efficacy of olanzapine and ziprasidone for the treatment of schizophrenia: a systematic review, *CNS Drugs* **19** (2005), 499–515.

[11] M.L. Michals, M.L. Crismon, S. Roberts and A. Childs, Clozapine response and adverse effects in nine brain-injured patients, *J Clin Psychopharmacol* **13** (1993), 198–203.

[12] Y. Mizoguchi, A. Monji, H. Isayama and N. Tashiro, Risperidone and refusal to eat after traumatic brain injury, *J Neuropsychiatry Clin Neurosci* **14** (2002), 87–88.

[13] W.J. Mysiw and M.E. Sandel, The agitated brain injured patient. Part 2: Pathophysiology and treatment, *Arch Phys Med Rehabil* **78** (1997), 213–220.

[14] R.L. Reyes, A.K. Bhattacharyya and D. Heller, Traumatic head injury: restlessness and agitation as prognosticators of physical and psychologic improvement in patients, *Arch Phys Med Rehabil* **62** (1981), 20–23.

[15] I. Richard, B. Perrouin-Verbe, J. Rome, C. Bernat and J.F. Mathe, Pharmacological treatment of post-traumatic behavioural disorders, *Ann Readapt Med Phys* **46** (2003), 49–57.

[16] F.R. Sallee, R. Kurlan, C.G. Goetz, H. Singer, L. Scahill, G. Law et al., Ziprasidone treatment of children and adolescents with Tourette's syndrome: a pilot study, *J Am Acad Child Adolesc Psychiatry* **39** (2000), 292–299.

[17] S. Schreiber, E. Klag, Y. Gross, R.H. Segman and C.G. Pick, Beneficial effect of risperidone on sleep disturbance and psychosis following traumatic brain injury, *Int Clin Psychopharmacol* **13** (1998), 273–275.

[18] B.V. Silver, L. Collins and K.A. Zidek, Risperidone treatment of motor restlessness following anoxic brain injury, *Brain Inj* **17** (2003), 237–244.

[19] S.M. Stahl and D.K. Shayegan, The psychopharmacology of ziprasidone: receptor-binding properties and real-world psychiatric practice, *J Clin Psychiatry* **64**(Suppl 19) (2003), 6–12.
